# Evaluation of Costimulatory Molecules in Peripheral Blood Lymphocytes of Canine Patients with Histiocytic Sarcoma

**DOI:** 10.1371/journal.pone.0150030

**Published:** 2016-02-22

**Authors:** Michihito Tagawa, Naoya Maekawa, Satoru Konnai, Satoshi Takagi

**Affiliations:** 1 Veterinary Medical Center, Department of Clinical Veterinary Science, Obihiro University of Agriculture and Veterinary Medicine, Obihiro, Hokkaido, Japan; 2 Department of Disease Control, Graduate School of Veterinary Medicine, Hokkaido University, Sappro, Hokkaido, Japan; 3 Department of Veterinary Clinical Sciences, Graduate School of Veterinary Medicine, Hokkaido University, Veterinary Teaching Hospital, Sappro, Hokkaido, Japan; Institut National de la Santé et de la Recherche Médicale (INSERM), FRANCE

## Abstract

Histiocytic sarcoma is a rapidly progressive and fatal neoplastic disease in dogs. It is unclear whether costimulatory molecules, including CD28, cytotoxic T-lymphocyte-associated antigen-4 (CTLA-4), and programmed death-1 (PD-1), are expressed on peripheral blood lymphocytes (PBLs) of canine patients with histiocytic sarcoma. The objective of this study was to evaluate the expression of CD28, CTLA-4, and PD-1 molecules on PBLs of patients with histiocytic sarcoma, patients with other tumors, and healthy controls. Twenty-six dogs were included in the study, with eight, ten, and eight dogs in the histiocytic sarcoma, other tumor, and healthy control groups, respectively. PBLs and serum were prospectively obtained from patients diagnosed histopathologically with histiocytic sarcoma, other tumors and healthy controls. The surface expression of CTLA-4, CD28, and PD-1 on T lymphocytes was examined using flow cytometric analysis. Serum samples were frozen at −30°C until serum interferon-γ (IFN-γ) was measured by enzyme-linked immunosorbent assay. The expression level of CTLA-4 on CD4+ lymphocytes was significantly higher in the histiocytic sarcoma group than in the control group. The expression of CTLA-4 on CD8+ lymphocytes was significantly higher in the histiocytic sarcoma group than in the other two groups. In addition, the expression of PD-1 on CD8+ lymphocytes was significantly higher in the histiocytic sarcoma group than in the control group. However, no significant differences in CD28 expressions and serum IFN-γ levels were observed. The present results provided evidence showing that the expression levels of CTLA-4 on both CD4+ and CD8+ lymphocytes and PD-1 on CD8+ lymphocytes in peripheral blood obtained from dogs with histiocytic sarcoma were upregulated. The overexpressions of CTLA 4 and PD-1 suggested that antitumor immunity may be suppressed in dogs with histiocytic sarcoma.

## Introduction

Canine histiocytic sarcoma is a rapidly progressive, fatal neoplastic disease that arises from dendritic cells [1]. Canine histiocytic sarcoma can be classified as localized or disseminated. Another form of histiocytic sarcoma, hemophagocytic histiocytic sarcoma, arises from macrophages [2]. These diseases in the histiocytic sarcoma complex are most frequently observed in middle-aged Bernese mountain dogs, Rottweilers, flat-coated retrievers, and golden retrievers [1]. The Pembroke Welsh corgi is also at high risk of histiocytic sarcoma in Japan [3]. Localized histiocytic sarcoma is commonly treated by surgery and/or radiotherapy; however, most cases eventually develop distant metastases to the lungs, lymph nodes, or abdominal viscera [4]. Despite systemic chemotherapy with lomustine or doxorubicin, canine histiocytic sarcoma is often associated with the acquisition of multidrug resistance, leading to poor prognosis [5, 6].

T-cell functions are regulated not just by the T-cell antigen-specific receptor but also by costimulatory molecules. These proteins belong to the B7-cluster of differentiation 28 (CD28) family, which includes CD28, cytotoxic T-lymphocyte-associated antigen-4 (CTLA-4), and programmed death-1 (PD-1), and play critical roles in costimulation [7]. Full activation of T cells requires the ligation of the CD28 receptor with B7 family members, i.e., B7-1 (CD80) or B7-2 (CD86), on antigen-presenting cells (APCs) [8]. CTLA-4, which is expressed on the surface of activated T lymphocytes, transmits signals inhibitory to T-cell activation by binding to the same B7 family ligands with a much higher affinity [8]. PD-1 is another negative regulatory molecule that is a member of the B7-CD28 family [9]. PD-1 binds to its ligands, programmed death ligands 1 and 2 (PD-L1 and PD-L2), and this interaction leads to T-cell deactivation or apoptosis [10].

Histiocytic sarcoma cells express various cell surface antigens [1, 11]. Previous results showed elevated mRNA expression of cell surface antigens, including MHC class II and CD86, in tumor tissue from dogs with histiocytic sarcoma [12]. In addition, some inflammatory markers, such as fibrinogen, ferritin, and C-reactive protein, have been reported to be increased in the serum of dogs with histiocytic sarcoma [13–15], and the expression of CD28, CTLA-4, and PD-1 is affected by a systemic inflammation induced by various neoplastic, infectious, and autoimmune diseases [10, 16–18]. Therefore, these costimulatory molecules in patients with histiocytic sarcoma may be affected by the systemic immune status induced by histiocytic sarcoma and may play a critical role in antitumor activity because the histiocytic sarcoma tumor tissue expresses CD86 molecules that can bind to CD28 and CTLA-4 [12].

The aim of this study was to evaluate the expression of costimulatory molecules, including CD28, CTLA-4, and PD-1, on peripheral blood lymphocytes (PBLs) of patients with histiocytic sarcoma, patients with other tumors, and healthy controls and to assess the immune status in dogs with histiocytic sarcoma.

## Materials and Methods

### Sample collection

Eight dogs with histiocytic sarcoma (histiocytic sarcoma group), ten dogs with other tumors (other tumor group), and eight clinically healthy controls (control group) were enrolled in this prospective study. Patients in the histiocytic sarcoma and other tumor groups were diagnosed by histopathological examination using excisional or biopsy samples in a veterinary teaching hospital at Hokkaido University between June 2014 and March 2015. Thoracic digital radiography in three views and abdominal ultrasonography were performed periodically to assess metastasis in all cases. Although two dogs in the histiocytic sarcoma group were treated with lomustine, progressive gross lesions were observed. Except for these two dogs, no patients were treated for histiocytic sarcoma or other tumors before tissue collection or experimentation. Dogs in the control group were age-matched healthy patients who came to the hospital for medical examinations. This study was approved by the Institutional Animal Care and Use Committees at the Graduate School of Veterinary Medicine, Hokkaido University.

### Cell preparation

Heparinized peripheral blood and serum were obtained from all patients and healthy controls. All heparinized blood samples were diluted with an equal volume of 0.9% saline, and PBLs were separated by Ficoll-Paque (Lymphoprep; Axis-Shield PoC AS, Oslo, Norway) density gradient centrifugation [19]. Following centrifugation at 800 × *g* for 20 min at room temperature, PBLs were collected and washed twice with 0.9% saline. The serum samples were stored at −30°C until use.

### Flow cytometric analysis

For flow cytometric analysis, cells were pelleted by centrifugation for 5 min at 1,500 rpm and washed with phosphate-buffered saline (PBS) containing 10% normal goat serum. Between 0.5 × 10^6^ and 1 × 10^6^ cells were stained for CD4, CD8, CD28, CTLA-4, and PD-1 for 30 min at 37°C. The following antibody conjugates were used: rat anti-canine CD4 mAb: YKIX302.9 prelabeled with fluorescein isothiocyanate; rat anti-canine CD8 monoclonal antibodies (mAbs): YCATE55.9 prelabeled with r-phycoerythrin (AbD Serotec, Raleigh, NC, USA) [20]; mouse anti-canine CD28 mAbs: 5B8 (Affymetrix, Santa Clara, CA, USA) [21]; mouse anti-human CTLA-4 mAbs: ANC152.2 (Ancell Corporation, Bayport, MN, USA); and goat anti-human PD-1 polyclonal antibodies (pAbs): BAF1086 (R&D Systems, Minneapolis, MN, USA) [22]. Additionally, dye/isotype-matched antibodies were included in all experiments as controls. After washing with PBS containing 10% goat serum, the cells were incubated with APC-conjugated anti-mouse IgG mAbs (Affymetrix) for CD28 and CTLA-4 and with APC-conjugated streptavidin (BioLegend, San Diego, CA, USA) for PD-1 for 30 min at 37°C. The cells were then washed and immediately analyzed using FACS Verse (BD Biosciences, San Jose, CA, USA) and FCS Express 4 (De Novo Software, Glendale, CA, USA). A minimum of 10,000 cells were analyzed for each sample tube and then progressively sorted by CD4 or CD8 positivity for subsequent analysis. Detection limits were set based on the isotype controls such that less than 1% of cells were positive.

The specificity of the anti-CTLA-4 antibody for canine CTLA-4 was confirmed by western blotting. In brief, concanavalin A-stimulated human and canine PBLs were lysed in 2× sodium dodecyl sulfate (SDS) sample buffer (ATTO, Tokyo, Japan) and boiled for 10 min. The samples were separated on 4–15% SDS-polyacrylamide gels and transferred onto a polyvinylidene difluoride membrane (Bio-Rad Laboratories, Hercules, CA, USA). After being blocked with EzBlock Chemi (ATTO), the membranes were incubated at room temperature for 1 h with mouse anti-human CTLA-4 antibodies, followed by washing with PBS containing 0.05% Tween 20 and incubation with horseradish peroxidase-conjugated goat anti-mouse IgG pAbs (Invitrogen, Carlsbad, CA, USA). After washing, the membrane was incubated with EzWestBlue (ATTO).

### Assessment of interferon-γ (IFN-γ) concentration

To assess IFN-γ concentrations, serum samples were thawed and analyzed by enzyme-linked immunosorbent assay with a Canine IFN-γ immunoassay kit (R&D Systems) according to the manufacturer’s instructions.

### Statistical analysis

All analyses were performed using JMP 11 (SAS Institute Inc., Cary, NC, USA). The sexes within each group were compared using the chi-squared test. Continuous variables, such as age and CD28, CTLA-4, and PD-1 parameters, were analyzed by the Mann-Whitney *U* tests for two groups or the Kruskal-Wallis test followed by the Steel-Dwass test for more than two groups. Spearman’s rank-correlation test was used to investigate the presence of a correlation between age and CD28 expression on CD8+ lymphocytes. Differences were considered statistically significant if the *P* value was less than 0.05.

## Results

### Characteristics of the study population

The distributions of selected characteristics of each group are presented in Table 1. The median ages of the groups were as follows: 9.5 years (range, 7–12 years) for the histiocytic sarcoma group, with three females (one intact and two spayed) and five males (all neutered); 12 years (range, 9–15 years) for the other tumor group, with four females (two intact and two spayed) and six males (two intact and four neutered); and 8.5 years (range, 5–15 years) for the control group, with one female (intact) and seven males (six intact and one neutered). There were no significant differences in age or sex between the three groups. In the histiocytic sarcoma group, four dogs were diagnosed with localized histiocytic sarcoma, and four dogs were diagnosed with disseminated histiocytic sarcoma. The other tumor group included the following tumors: malignant melanoma, squamous cell carcinoma, pulmonary adenocarcinoma, hemangiosarcoma, osteosarcoma, transitional cell carcinoma (nonpapillary/infiltrating type), soft tissue sarcoma, and anal sac adenocarcinoma (Table 1). Lung, lymph node, or abdominal organ metastases were detected at the time of the diagnosis in five of the ten dogs in the other tumor group.

**Table 1:** Clinical information of samples.

Case (n)	Breed (n)	Age* (range)	Sex (n)	Diagnosis (n, sites)
**HS (8)**	WC (3)	9.5 (7–12)	F (3)	Localized HS (4)
	FCR (2)		M (5)	(2, brain; 1, lung; 1, skin)
	Other (3)			Disseminated HS (4)
**Other tumor (10)**	BMD (2)	12 (9–15)	F (4)	TCC (2, bladder)
	MD (2)		M (6)	MM (2, mandible)
	Other (6)			SCC (1, mandible)
				PAC (1, lung)
				HSA (1, spleen)
				OSA (1, mandible)
				STS (1, subcutaneous)
				ASA (1, anal sac)
**Control (8)**	Beagle (2)	8.5 (5–15)	F (1)	-
	BMD (2)		M (7)	
	Other (4)			

HS, histiocytic sarcoma; BMD, Bernese mountain dog; WC, Welsh corgi; FCR, flat-coated retriever; MD, miniature dachshund; F, female; M, male; TCC, transitional cell carcinoma; MM, malignant melanoma; SCC, squamous cell carcinoma; PAC, pulmonary adenocarcinoma; HAS, hemangiosarcoma; OSA, osteosarcoma; STS, soft tissue sarcoma; ASA, anal sac adenocarcinoma.

*: median (years)

### CD28, CTLA-4, and PD-1 expression on peripheral lymphocytes

**Fig 1:**
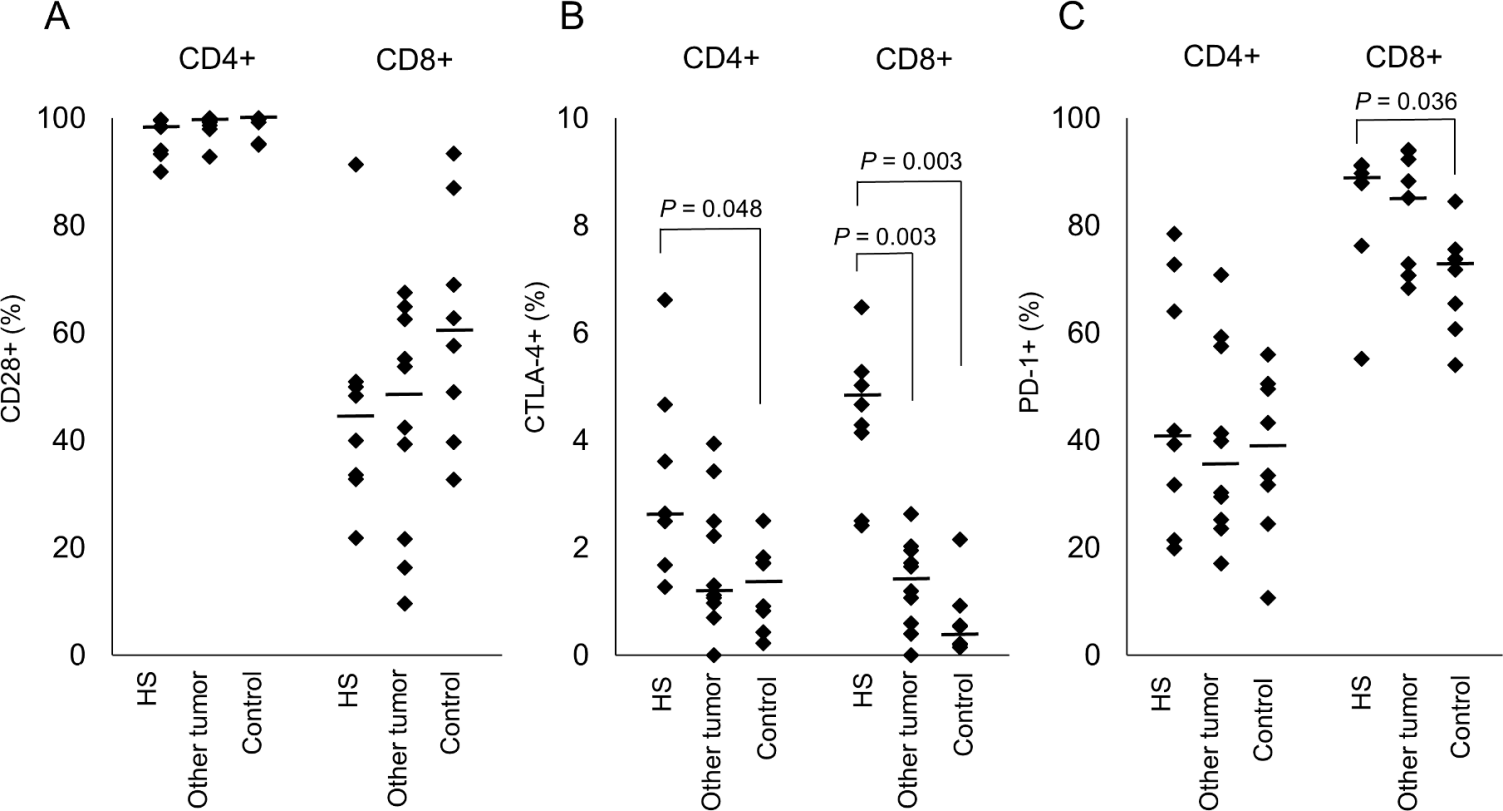
Percent expression of each costimulatory molecule in CD4+ and CD8+ lymphocytes. CD28 (A), CTLA-4 (B) and PD-1 (C) expression on CD4+ and CD8+ T cells in the peripheral blood of histiocytic sarcoma, other tumor, and control groups. Each dot represents a patient and the bar represents the median. *P*-values are shown. HS, histiocytic sarcoma.

After confirming cross-reactivity using the anti-CTLA-4 antibodies (S1 Fig.), the expression of each costimulatory molecule on cells was calculated as the percentage of CD28+, CTLA-4+, or PD-1+ cells per CD4+ and CD8+ lymphocyte gate for the three groups (S2 Fig.). There were no significant differences in CD28 expression on CD4+ and CD8+ lymphocytes between groups (Fig 1A). The expression level of CTLA-4 on CD4+ lymphocytes was significantly higher in the histiocytic sarcoma group (mean ± standard deviation: 3.20% ± 1.74%) than in the control group (1.27% ± 0.79%; *P* = 0.048). The expression of CTLA-4 on CD8+ lymphocytes was also significantly higher in the histiocytic sarcoma group (4.35% ± 1.37%) than in the other tumor group (1.32% ± 0.82%; *P* = 0.003) and the control group (0.62% ± 0.68%; *P* = 0.003; Fig 1B). In addition, the expression of PD-1 on CD8+ lymphocytes was significantly higher in the histiocytic sarcoma group (83.83% ± 12.59%) than in the control group (69.96% ± 9.53%; P = 0.036; Fig 1C). In addition, there were no correlations between age and CD28 expression on CD8+ lymphocytes among all the dogs (Spearman’s rank-correlation coefficient: -0.136, *P* = 0.509).

CD28, CTLA-4, and PD-1 expression levels were also compared between four dogs with localized histiocytic sarcoma and four dogs with disseminated histiocytic sarcoma in the histiocytic sarcoma group and five dogs with metastasis and five dogs without metastasis in the other tumor group. Although there were no significant differences for all parameters between dogs with localized and disseminated histiocytic sarcoma (Table 2), CTLA-4 expression on CD8+ T cells was significantly higher in dogs with metastasis than in dogs without metastasis (Table 3).

**Table 2:** Comparison of costimulatory molecules in dogs with localized and disseminated histiocytic sarcoma.

Costimulatory molecules	Lymphocyte subset	Localized HS (n = 4)	Disseminated HS (n = 4)	*P*-value
**CD28**	CD4+	97.8 ± 3.0	95.5 ± 4.4	0.471
	CD8+	45.7 ± 8.2	46.5 ± 30.7	0.471
**CTLA-4**	CD4+	3.1 ± 2.4	3.4 ± 1.0	0.665
	CD8+	4.1 ± 1.1	4.6 ± 1.7	0.665
**PD-1**	CD4+	28.7 ± 10.2	63.6 ± 17.3	0.061
	CD8+	87.2 ± 7.3	80.5 ± 17.0	0.312

*P*-values in bold are statistically significant (*P* < 0.05). Mean±SD (%). HS, histiocytic sarcoma.

**Table 3:** Comparison of costimulatory molecules in dogs in the other tumor group with or without metastasis.

Costimulatory molecules	Lymphocyte subset	With metastasis (n = 5)	Without metastasis (n = 5)	*P*-value
**CD28**	CD4+	99.4 ± 0.5	97.9 ± 2.9	0.753
	CD8+	46.5 ± 18.7	40.1 ± 25.1	0.835
**CTLA-4**	CD4+	2.6 ± 1.1	0.8 ± 0.5	0.060
	CD8+	2.0 ± 0.4	1.0 ± 0.5	**0.012**
**PD-1**	CD4+	35.9 ± 19.7	43.0 ± 17.0	0.531
	CD8+	79.2 ± 12.8	85.1 ± 7.8	0.401

*P*-values in bold are statistically significant (*P* < 0.05). Mean±SD (%)

### IFN-γ concentration

The IFN-γ concentration in the serum obtained from each dog was determined. The concentration of IFN-γ in the serum was lower in the histiocytic sarcoma group (30.82 ± 16.35 pg/mL) than in the other tumor group (73.41 ± 61.91 pg/mL); however there were no significant differences between the three groups (control group: 30.63 ± 25.65 pg/mL).

## Discussion

Costimulatory molecules, including CD28, CTLA-4, and PD1, act as the main immune components regulating immune responses [9]. Recently, various studies have focused on the interactions of CTLA-4 or PD-1 with their ligands because of their role in regulating adaptive immunity [23, 24]. Blockade of CTLA-4 or PD-1 results in pronounced antitumor activity, and monoclonal antibody therapies against these targets improve overall survival in patients with various neoplasias [25, 26]. In particular, CTLA-4 may play an important role in antitumor activity in canine histiocytic sarcoma because of the high expression of the ligand CD86, which can bind to CTLA-4 [12].

In the present study, the expression levels of these immunological molecules were determined in peripheral lymphocytes obtained from canine patients with histiocytic sarcoma, other tumors, and healthy controls. There were no significant differences in CD28 expression between the groups. CD28 is expressed on the surface of most CD4+ and half of CD8+ adult human peripheral blood T cells [27]. In addition, the CD8+ subset shows progressively decreasing CD28 expression with age [28]. However, in this study, there were no correlations between age and CD28 expression on CD8+ lymphocytes among all the dogs. It may be necessary to examine CD28 expression on CD8+ lymphocytes obtained from dogs of various ages because the ages of dogs in this study were restricted.

CTLA-4 expression was significantly increased on CD4+ and CD8+ lymphocytes in the histiocytic sarcoma group. The expression of CTLA-4 on T cells depends on cell activation induced by the CD28–B7 interaction, and systemic inflammatory diseases, such as infectious or autoimmune diseases, induce high expression of CTLA-4 [16, 17]. Previous studies have shown that a significant inflammatory response is mounted in association with histiocytic sarcoma in dogs, and this could be associated with the chronic inflammatory response or necrotic foci of a large tumor burden [13, 14]. Moreover, the systemic immune status in dogs with histiocytic sarcoma may be affected by the severe inflammatory conditions involved in local infiltration of histiocytic sarcoma. Regulatory T cells, which express CD4, CD25, and Foxp3, are known to express CTLA-4 constitutively, and CTLA-4 plays a functionally significant role in the suppression of regulatory T cells [29]. Thus, the overexpression of CTLA-4 on CD4+ cells obtained in this study may be associated with increased numbers of regulatory T cells. In contrast, the presence of CD8+ lymphocytes, the main tumor killers in specific immunity, showing overexpression of CTLA-4 indicated that antitumor immunity may be suppressed in dogs with histiocytic sarcoma. A CTLA-4 blockade may induce restoration of antitumor immunity against histiocytic sarcoma. In addition, there was no significant difference in levels of CTLA-4 expression between dogs with localized histiocytic sarcoma and disseminated histiocytic sarcoma. Therefore, the expression of CTLA-4 on peripheral lymphocytes may not be influenced by the tumor type of the histiocytic sarcoma. Recently, a challenging strategy of using antibodies to block the CTLA-4 molecule has emerged, and several studies have demonstrated the effects of CTLA-4 blockade on the induction of tumor immunity and the rejection of tumors not only in animal models but also in human patients [30, 31]. In particular, ipilimumab, which blocks CTLA-4, has improved overall survival in patients with metastatic melanoma [32]. The results of the present study provide evidence that such immunotherapy regimens may also be applicable in canine histiocytic sarcoma.

PD-1 plays an important role in tumor immunity, and blockade of PD-1 could restore antitumor immunity to accelerate tumor eradication in various tumors [24]. In the present study, PD-1 expression was significantly increased on CD8+ lymphocytes in the histiocytic sarcoma group in comparison with that in the control group. In human medicine, a previous report demonstrated that upregulation of PD-1 on CD8+ T cells is related to interleukin (IL)-10 and IL-6 [33]. However, these cytokines are not upregulated in dogs with histiocytic sarcoma [13]. Although the mechanisms responsible for PD-1 upregulation in histiocytic sarcoma remain unclear, the results of the present study suggest that PD-1 may become a new therapeutic target for histiocytic sarcoma in dogs. In addition, PD-1 is primarily involved in modulating T-cell activity in peripheral tissues via its interactions with PD-L1 and PD-L2 [34]. PD-1 expression is upregulated on tumor-infiltrating lymphocytes, and this may also contribute to tumor immunosuppression [35]. A past study revealed that PD-1 expression on CD4+ and CD8+ T cells from gastric cancer tissue was significantly higher than that on CD4+ and CD8+ T cells from PBLs [33]. Further studies are needed to evaluate PD-1 expression on tumor-infiltrating lymphocytes.

Despite there were no significant differences between the groups, serum IFN-γ concentrations were lower in the histiocytic sarcoma group than in the other tumor group. IFN‑γ is released by Th1 subgroup cells and has antitumor effects [36]. Thus, antitumor immunity may be decreased in dogs with histiocytic sarcoma in comparison with dogs having other tumors.

In human medicine, peripheral lymphocytes of patients with some cancers, including lung cancer, breast cancer, and leukemia, exhibit increased expression of the CTLA-4 protein or gene [37–39]. An increase in CTLA-4-expressing lymphocytes was observed in some dogs in the other tumor group and was related to metastasis status in that group. Moreover, CTLA-4 expression on peripheral lymphocytes may behave as a prognostic factor in some tumors. Additional large-scale studies are needed to evaluate which tumors are associated with overexpression of CTLA-4.

In conclusion, the present study demonstrated upregulation of CTLA-4 expression on both CD4+ and CD8+ T cells and PD-1 expression on CD8+ T cells in peripheral blood obtained from dogs with histiocytic sarcoma. Overexpressions of CTLA-4 and PD-1 suggested suppression of antitumor immunity in dogs with histiocytic sarcoma; these molecules may represent new therapeutic targets for the treatment of canine histiocytic sarcoma.

## Supporting Information

S1 Fig Confirmation of the cross-reactivity of the anti-CTLA-4 antibody by western blotting. M, protein marker.
(TIF)

S2 Fig Analysis of a PBL sample for CD28, CTLA-4, and PD-1 expression in the CD4+ lymphocyte gate. A representative sample of forward versus side scatter identified the predominant lymphocyte population captured in region P1. The proportions of CD4 (P3) and CD8 (P2) cells in P1 are indicated (A). The proportions of CD28 (B), CTLA-4 (C), and PD-1 (D) expression cells in P3 are indicated in the middle panels. The left panels show each isotype control, and the right panels show histograms of lymphocyte gated cells. SSC, side scatter; FSC, forward scatter; PE, phycoerythrin; FITC, fluorescein isothiocyanate; APC, allophycocyanin.
(TIF)
