# Supplementary figures and images for: Evaluation of Costimulatory Molecules in Peripheral Blood Lymphocytes of Canine Patients with Histiocytic Sarcoma
Source: PLoS One. 2016 Feb 22;11(2):e0150030. doi: 10.1371/journal.pone.0150030 (PMC4767183; doi:10.1371/journal.pone.0150030)

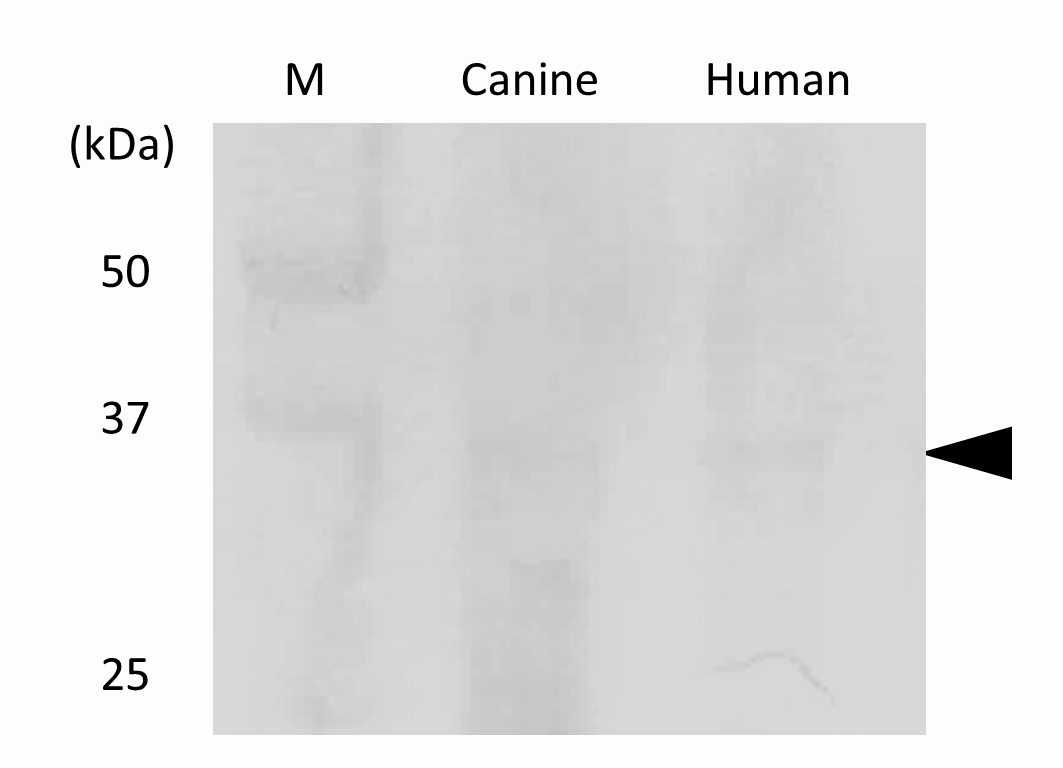

Supplement: S1 Fig — M, protein marker. (TIF) [file pone.0150030.s001.TIF]

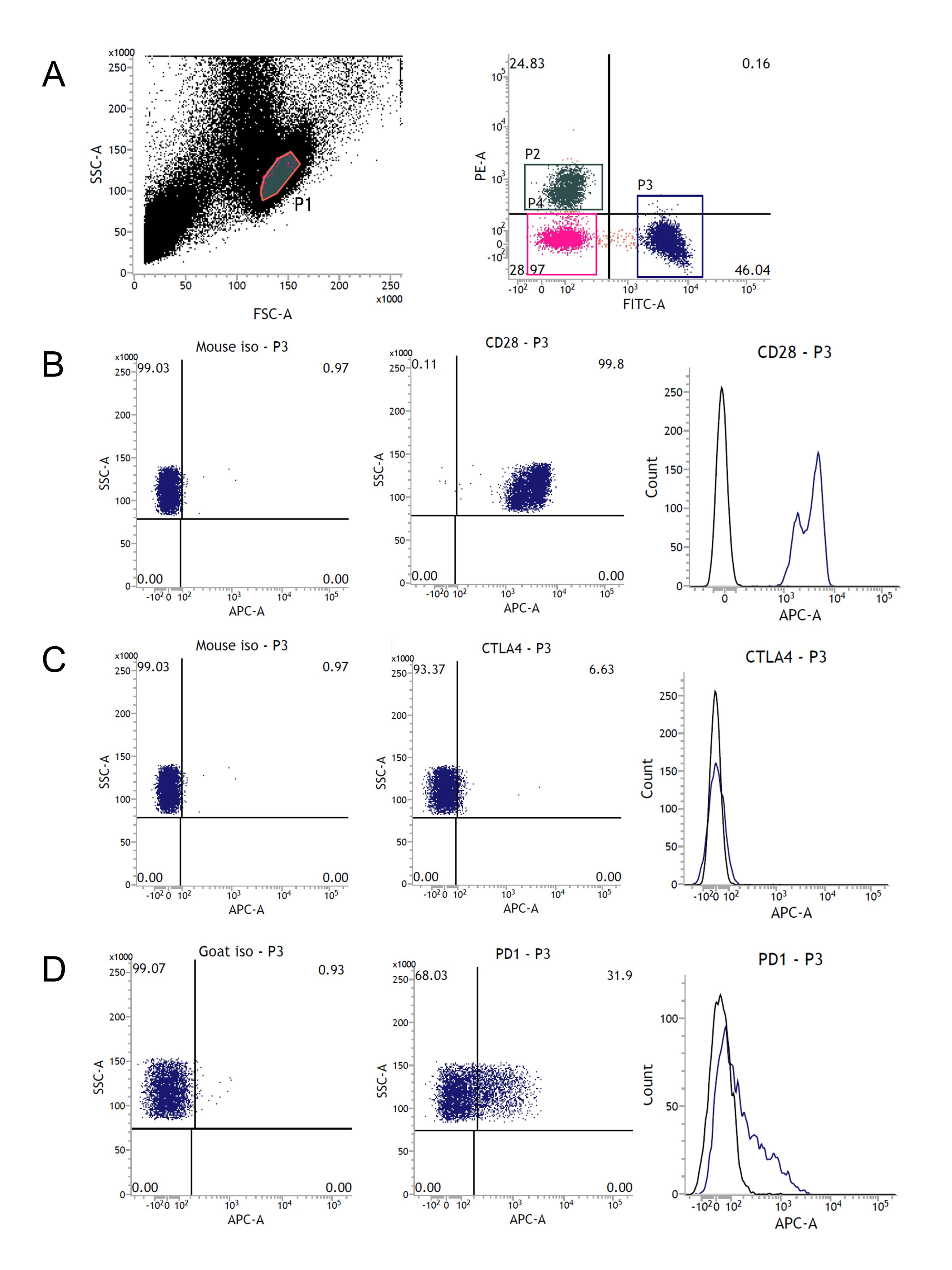

Supplement: S2 Fig — A representative sample of forward versus side scatter identified the predominant lymphocyte population captured in region P1. The proportions of CD4 (P3) and CD8 (P2) cells in P1 are indicated (A). The proportions of CD28 (B), CTLA-4 (C), and PD-1 (D) expression cells in P3 are indicated in the middle panels. The left panels show each isotype control, and the right panels show histograms of lymphocyte gated cells. SSC, side scatter; FSC, forward scatter; PE, phycoerythrin; FITC, fluorescein isothiocyanate; APC, allophycocyanin. (TIF) [file pone.0150030.s002.tif]
